# Influence of Si, Cu, B, and Trace Alloying Elements on the Conductivity of the Al-Si-Cu Alloy

**DOI:** 10.3390/ma15020426

**Published:** 2022-01-06

**Authors:** Zhao Yang, Xiaolong He, Bin Li, Andrej Atrens, Xuyue Yang, Hanming Cheng

**Affiliations:** 1 School of Materials Science and Engineering, Central South University, Changsha 410083, China; wangwz@csu.edu.cn (X.H.); lx1122@csu.edu.cn (B.L.); yangxuyue@csu.edu.cn (X.Y.); 2 School of Mechanical and Mining Engineering, The University of Queensland, Brisbane, QLD 4072, Australia; andrejs.atrens@uq.edu.au; 3 Nantong Hongjin Aluminum Investment Co., Ltd., Nantong 226300, China; chm@huajin-group.com

**Keywords:** aluminum alloy, ADC12, conductivity, Al-B master alloy, micro-alloying

## Abstract

The influence of Si, Cu, B, and trace alloying elements on the conductivity of aluminum die cast 12 (ADC12) alloy was investigated. The conductivity decreased linearly with increasing volume fraction of the Si phase attributed to a linear decrease of the volume of the more conductive Al phase through a rule of mixtures. The conductivity also decreased with increasing Cu content, between 0~3%. The conductivity increased with increasing B content, reached the peak at 0.02% B and thereafter decreased somewhat. The mechanism was that B reacted with the transition element in the Al phase to form boride, decreasing the transition element concentration in the Al lattice, and decreasing the lattice constant. The thermal conductivity, λ, was related to the electrical conductivity, σ, by means of $\lambda =\mathrm{LT}\sigma +\lambda_{g}$, where L is the apparent Lorentz constant, 1.86 × 10^−8^; T is the absolute temperature, 293 K; λ_g_ is the lattice conductivity, 42.3 W/(m·K).

## 1. Introduction

The direct industrial importance of conductivity in aluminum alloys is best emphasized by their extensive use as electrical conductors. Aluminum die cast 12 (ADC12) alloy is a eutectic aluminum–silicon (Al-Si) alloy containing 10–12 wt.% Si. The major alloying elements are Si and Cu and trace alloying elements include Ti, Mn, V, and Cr [1,2]. ADC12 is a popular pressure die-casting Al alloy because of a good combination of castability, productivity, corrosion resistance, wear resistance, mechanical properties, low price, and environment friendliness [3,4,5,6,7,8]. ADC12 has wide commercial applications such as LED lamp sockets, radiators in wireless communication stations, indoor heating, and parts in the automotive industry like the engine blocks and cylinder liners [9,10]. The power of these components has been increasing quickly, which requires an increased efficiency of heat withdrawal and requires ADC12 with increased conductivity [11,12]. Better future prospects of ADC12 thus require a version with improved conductivity.

The conductivity of Al-Si alloys has been extensively researched in the last few decades [13,14,15,16,17,18,19]. Wen et al. [13] found that high conductivity in Al-7Si alloys was produced through the optimization of the eutectic Si: i.e., by changing the size and distribution of the eutectic Si phase by a transformation from coarse plates to fine short slabs or coral-shaped particles. Sigworth [14] investigated the influence of Si morphology on conductivity. Mulazimoglu et al. [15] and Shin et al. [16] found that the conductivity of the Al-Si alloy decreased linearly with increasing Si content and with the volume of the Si phase. Li et al. [17] found increased conductivity in an Al-Si binary alloy when heat treatment broke up the acicular-shaped eutectic Si particles into smaller segments and caused gradual spheroidization. Belov et al. [18] found a lower conductivity for a higher Cu content attributed to more lattice distortion. Jiang et al. [19] found that the soluble alloying elements like Cu decreased conductivity of the primary alpha Al phase and that these could form intermetallic compounds and could also change other kinds of compounds. These studies have indicated that the conductivity of Al-Si alloys depends on the major alloying additions. Table 1 lists the most commonly used commercial high conductivity Al-Si Alloys. It can be seen that the variation of Si content and Cu content is one of the major factors that determines the conductivity and the mechanical properties. However, the mechanism by which these major alloying additions influence the conductivity needs to be further clarified. Furthermore, trace elements in ADC12 have not received the same attention, and little research has been conducted to investigate their influence on the conductivity of Al-Si alloys. Trace alloying elements are inevitable in industrial-grade pure aluminum ingots [20], whose total trace-element content varies from 100 to 400 parts-per-million (ppm). In order to eliminate the potential negative influence of trace alloying elements on the conductivity of Al cables, an attempt is made to convert these trace elements to borides by adding Al-boron (Al-B) ingots into the AA1XXX or AA6XXX molten metal before casting [21,22,23,24,25,26]. However, the morphology and distribution of borides in Al-Si alloys, and their influence on conductivity of Al-Si alloy are not yet very clear.

This work studied the influence of (i) Si, Cu, and trace alloying elements, and (ii) the addition of Al-B master alloy on the conductivity of ADC12, paying special attention on the morphology and distribution of boride and their influence mechanism on the conductivity. The crystal distortion method during X-ray flaw detection was creatively used to characterize conductivity of ADC12.

## 2. Experimental Procedures

The ADC12 alloys were made using commercial AA1070 aluminum ingots (Baotou Aluminum Co., Ltd, Baotou, China), 441 silicon ingots (Hebi Xinyi metal material Co., Ltd, Hebi, China) and C10200 copper ingots(Yunnan Copper Industry Co., Ltd, Kunming, China). The commercial Al-4%B master alloy was added to the melt. The compositions of the raw materials and typical Al-Si-Cu alloys are presented in Table 2. Samples were melted in a resistance furnace using a graphite–clay crucible. Molten aluminum was heated to 800 °C, then Si, Cu and B were added. After purification and degassing using 5N purity Ar gas, the melt was poured into a steel mold at 200 °C. The outline and the size of the test bars coincided with ASTM standard E8 [27]. The diameter of the gage part was 6.4 ± 0.1 mm. The length of the gage part was 60 mm.

Electrical conductivity was measured using a commercial high-resolution micro-ohm gauge (Zhengyang 9510, Shanghai, China) at room temperature. The tensile test bars were measured by a double Wheatstone Bridge in the micro-ohm gauge. The thermal conductivity was measured using an AnterFlashline 3000 S2 laser thermal gauge (Anter Cooperation, Pittsburgh, PA, USA); the test samples had a diameter of 12 mm and a thickness of 3 mm. Mechanical properties were measured using a CSS-44100 tensile test machine (Changchun Experimental Machine Co., Ltd, Changchun, China) at a speed of 2 mm/min. Microstructures were characterized using an OLYMPOUS-PMG3 optical microscope (Olympus (Shenzhen) Industry Co., Ltd, Shenzhen, China) and a Quanta MK2-200 scanning electron microscope (SEM, FEI Company, Eindhoven, The Netherlands) equipped with an energy-dispersive X-ray spectrometer (EDS). The lattice parameters were measured using a D/max-2500/PC X-ray diffraction (XRD, Rigaku Corporation, Tokyo, Japan): the diffraction rated power 3 kW, Cu target, tube voltage 35 kV, incident angle ranged from 10° to 80°. The Al(220) XRD peaks had a little more shape than the Al(311) peaks. The lattice constants of different Al-Si-Cu alloys were analyzed using the software program Jade 5.0 (Materials Data Inc., California, USA). The trace elements were investigated using the JXA-8230 Electron Probe Micro-analysis (EMPA, Japan Electronics Corporation (JEOL), Tokyo, Japan). Transmission electron microscopy (TEM) was observed on a Tecnai G2 20ST system (FEI Company, Hillsboro, OR, USA).

## 3. Results

Table 3 presents the electrical, thermal, and mechanical properties of the samples with different compositions, in which the composition deviation of Si and Cu was controlled within 0.2 wt.%. The influence of Si and Cu on the conductivity was significant as shown in Table 3. For the cases of zero Cu content, increasing Si content caused a decrease of electrical and thermal conductivities. Similarly, for the constant Si content of 9.8%, increasing Cu content decreased the electrical and thermal conductivities. Addition of 0.02% B increased the electrical conductivity by more than 10%. When the Si content was lower than the eutectic concentration, increasing Si content increased the tensile strength. Increased Cu content up to 3% increased strength but a greater Cu decreased mechanical properties. A boron content less than 0.05% had little influence on the mechanical properties. The T6 heat-treatment significantly increased the electrical conductivity, thermal conductivity, and the mechanical properties. The thermal conductivity of as-cast Al-9.8Si-2Cu-0.02B was 146.2 W/(m·K) and was 166.7 W/(m·K) after the T6 heat-treatment.

**Figure 1:**
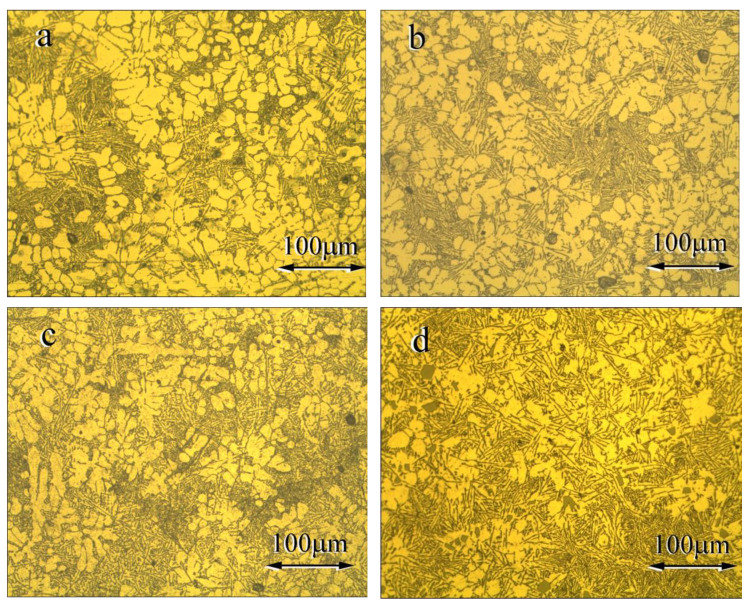
Microstructures of the as-cast Al-Si alloys containing the following Si contents: (**a**) 9.8% Si; (**b**) 10.8% Si; (**c**) 11.8%Si; and (**d**) 12.8% Si.

Figure 1 presents the microstructures of the as-cast Al-Si alloys. The Si content of these bars varied around the eutectic composition. Figure 1a illustrates the microstructure of Al-9.8Si alloy, which was composed of the dendritic primary Al (α) phase and the needle like eutectic micro-structure anomalously orientated. Figure 1b presents the microstructure of Al-10.8Si, which had an increased volume of the eutectic microstructure and a small amount of primary crystalline silicon. Figure 1c,d present the microstructures of Al-11.8Si and Al-12.8Si, which had increasing volumes of the eutectic microstructure and decreasing volumes of the primary Al phase. Furthermore, a large amount of eutectic Si destroyed the continuity of the Al matrix (Figure 1d).

**Figure 2:**
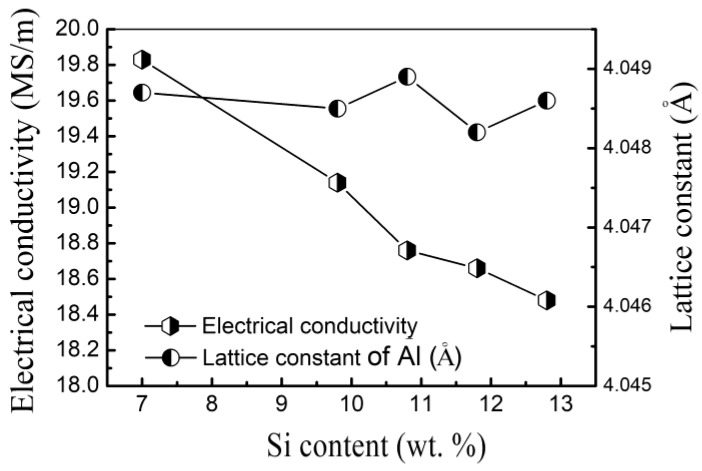
Relationship between Si content and electrical conductivity, and lattice constant.

**Figure 3:**
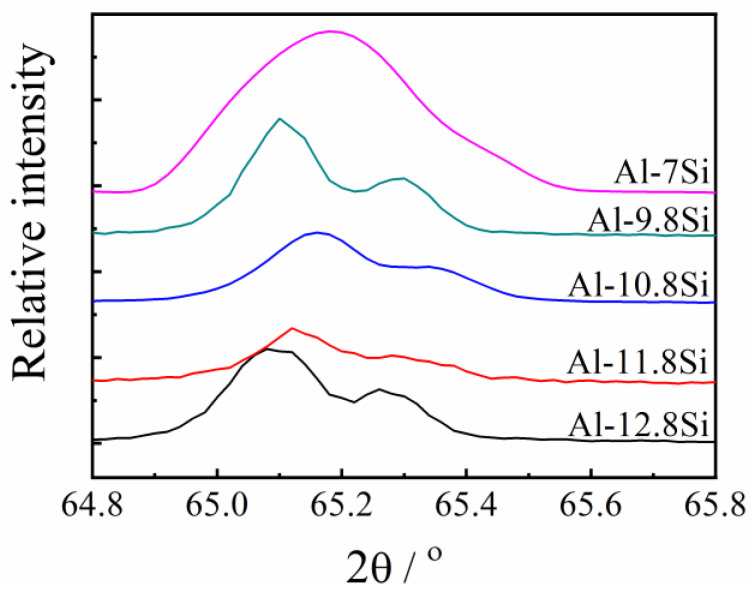
X-ray diffraction spectra of the Al-Si alloys with the stated Si contents.

Figure 2 presents the relationship between the Si content and the electrical conductivity and the lattice constants of the Al-Si alloys. The as-cast Al-7% Si had the highest electrical conductivity, 19.83 MS/m; and Al-12.8% Si had the lowest electrical conductivity, 18.48 MS/m. The increase of Si content from 7% and 12.8% linearly decreased the electrical conductivity, whereas the lattice constant fluctuated within the range from 4.0480 Å to 4.0489 Å. The effect of Si content on the lattice distortion was not significant. Representative XRD results are presented in Figure 3.

**Figure 4:**
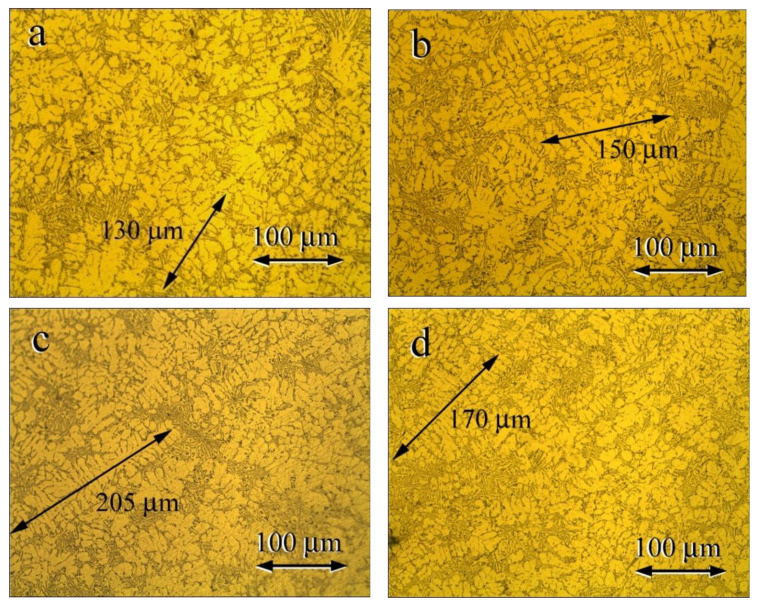
Microstructures of as-cast Al-Si-Cu alloys with the following Cu contents: (**a**) 0.2% Cu; (**b**) 1% Cu; (**c**) 2% Cu; and (**d**) 3% Cu.

Figure 4 presents the microstructures of the as-cast Al-Si-Cu alloys. These microstructures were composed of the dendritic primary (Al) phase and the needle like eutectic structure anomalously orientated, just like the as-cast Al-Si alloys. A high Cu content resulted in longer coarser primary Al dendrites attributed to enhanced dendrite growth due to enhanced constitutional supercooling caused by the high alloy [28].

**Figure 5:**
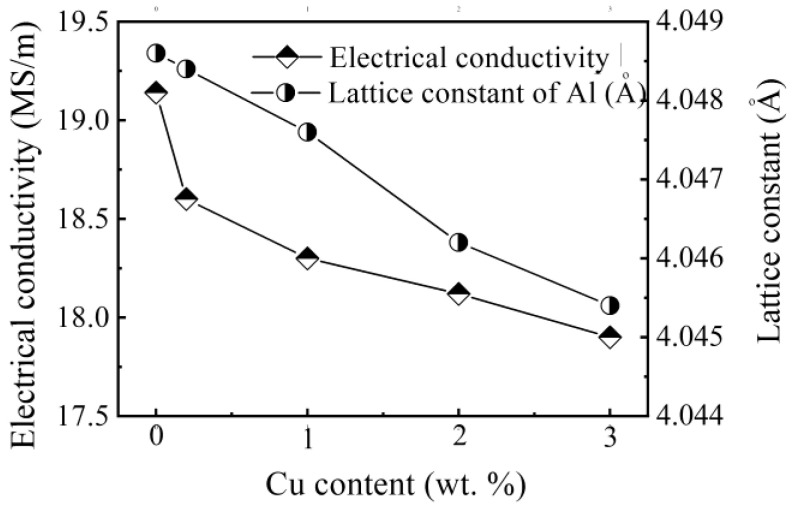
Relationship between Cu content and electrical conductivity, and lattice constants.

**Figure 6:**
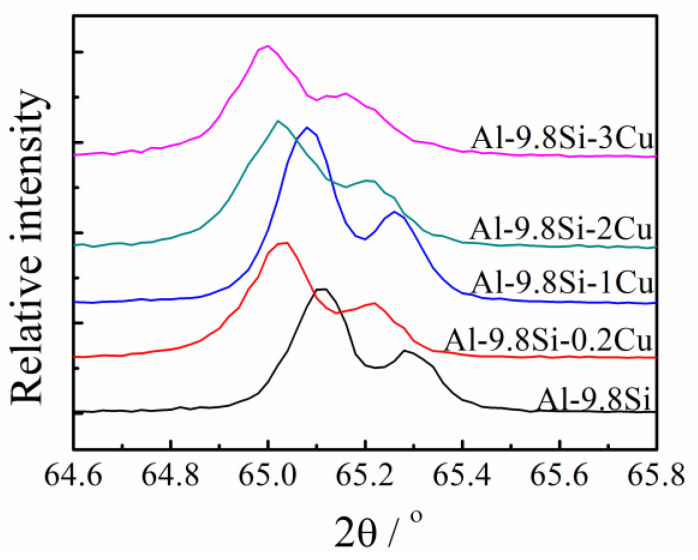
X-ray diffraction spectra of the Al-Si-Cu alloys with different Cu content.

Figure 5 presents the dependence of the electrical conductivity and the lattice constant on the Cu content. The electrical conductivity of the test bars with zero Cu content is the highest, 19.14 MS/m, and the corresponding lattice constant is 4.0486 Å, which is close to the pure Al lattice constant (4.0494 Å). The alloy containing 3% Cu had the lowest conductivity, 17.90 MS/m, and the smallest lattice constant of 4.0454 Å, which deviates the most from the lattice constant of pure Al. Increasing Cu content decreased the conductivities and the lattice constant. Representative X-ray diffraction (XRD) results are presented in Figure 6.

**Figure 7:**
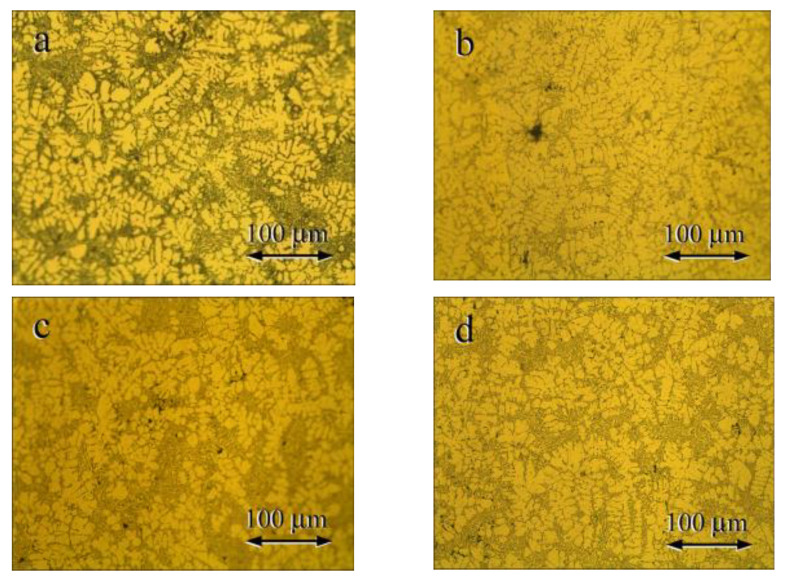
Microstructures of the as-cast Al-10 Si wt.% alloys with the following B contents: (**a**) 0.01 wt.%; (**b**) 0.02 wt.%; (**c**) 0.03 wt.%; and (**d**) 0.05 wt.%.

**Figure 8:**
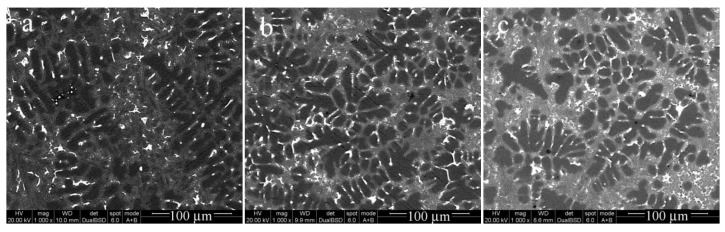
SEM microstructures of as-cast Al-9.8% Si-2% Cu alloys with B contents of (**a**) 0 wt.%; (**b**) 0.02 wt.%; and (**c**) 0.05 wt.%.

Figure 7 presents the microstructures of the as-cast Al-9.8%Si-2%Cu alloys containing the following B contents of (a) 0.01 wt.%, (b) 0.02 wt.%, (c) 0.03 wt.%, and (d) 0.05 wt.%. The microstructures of the B-containing Al-9.8%Si-2%Cu alloys had a primary Al phase refined compared with that of the B-free Al-9.8%Si-2%Cu alloy in Figure 4c. The grain size of the primary Al phase decreased dramatically and became equiaxial. This grain refinement agreed with the study of Murty [29], which found that B refined the primary Al phase of an Al-Si alloy. The microstructure was characterized by SEM using backscatter images to allow evaluation of the influence of the volume fraction of the eutectic microstructure on the conductivity. Figure 7 shows that the primary Al phase was refined with increasing B contents. Figure 8a–c presents the microstructures of the Al-9.8%Si-2%Cu alloy containing B contents of 0 wt.%, 0.02 wt.%, and 0.05 wt.%. Figure 8 indicated that the B refined the grain size and increased the volume of the eutectic structure.

**Figure 9:**
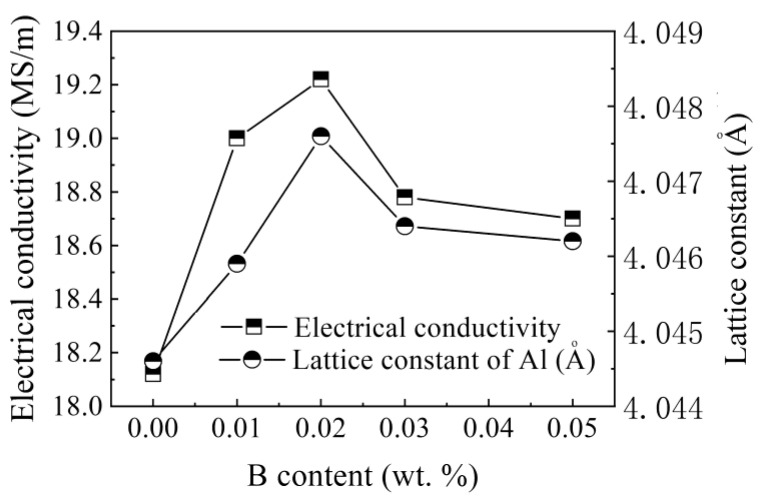
Relationship between B content and electrical conductivity, and lattice constants.

**Figure 10:**
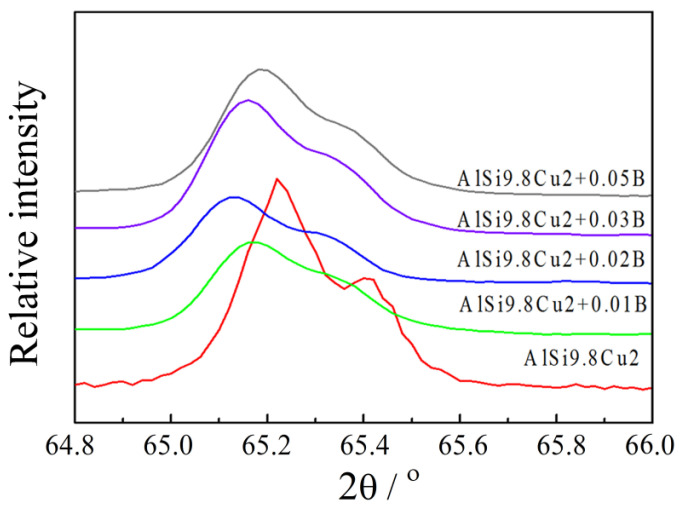
X-ray diffraction spectrums of the Al-9.8%Si-2%Cu aluminum alloys with different B contents.

Figure 9 presents the relationship of electrical conductivity and the lattice constants on the B content. The alloy with zero B content had the lowest conductivity, 18.48 MS/m, and the lowest lattice constant of 4.0463 Å, which was the most deviated from the pure Al lattice constant (4.0494 Å). Increasing B (0.01 wt.%) increased the electrical conductivity sharply to 19.00 MS/m for 0.01 wt.% B, and to a maximum of 19.22 MS/m for 0.02 wt.% B. Further increasing B contents caused some decrease of the electrical conductivity. Similarly, the lattice constant also increased with increasing B content, reached the maximum at 0.02% B and decreased slightly as the B content further increased. These results also shows that the greater deviation of the lattice constant from the pure Al lattice constant (4.0494 Å) correlated with lower the conductivity. Representative X-ray diffraction (XRD) results are presented in Figure 10.

B is a more critical element for high conductivity Al-Si-Cu alloy than Si and Cu. A small amount of B significantly increases the conductivity. B element also refined the primary Al phase, which is important in eliminating casting defects. B did not affect the mechanical properties. Increasing Si content did not influence the lattice constant of the Al phase, but decreased the electrical conductivity. Increasing Cu content decreased the lattice constant of the Al phase and decreased the electrical conductivity. Obviously, B has more comprehensive influence on the electrical conductivity.

## 4. Discussion

### 4.1. Relationship between Thermal Conductivity and Electrical Conductivity

The measurement of electrical conductivity of an alloy is typically much easier than the measurement of the thermal conductivity. A convenient method to evaluate the thermal conductivity of Al-Si-Cu products is important to the manufactures. A good solution is to evaluate the thermal conductivity from the measurement of electrical conductivity.

The principal carriers of heat in metals are electrons and lattice waves, which allow the thermal conductivity to be written as:

(1) $$\lambda ={\;\lambda }_{e}+\lambda_{g}$$

where λ_e_ is the electronic conductivity and λ_g_ the lattice conductivity. The thermal conductivity (λ) is also related to the electrical conductivity according to the Wiedemann–Franz law:

(2) $$\lambda =\mathrm{LT}\sigma +\lambda_{g}$$

where L is the Lorentz constant, T the temperature, and σ the electrical conductivity [15,30,31]. As L is almost a constant in the Al-Si-Cu system, the electrical conductivity can be used to evaluate the thermal conductivity of the ADC12 alloys.

**Figure 11:**
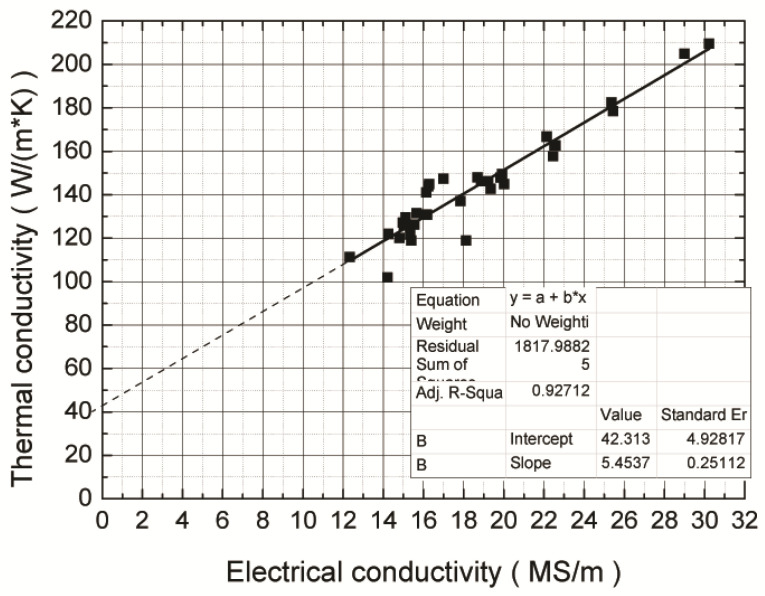
The relationship between electrical conductivity and thermal conductivity of the Al-Si-Cu alloys.

Table 3 presents the thermal conductivity of typical alloys. Figure 11 presents the measured relationship between thermal conductivity and electrical conductivity. The thermal conductivity was linearly related to the electrical conductivity, consistent with Equation (2). Statistical analysis indicated that the value of λ_g_ was 42.3 W/(m·K) and the measured Lorentz constant was 1.86 × 10^−8^ at room temperature at 293 K. 

It is noteworthy that Equation (2) provided the relationship between thermal conductivity and electrical conductivity for all the specimens, including the as cast specimens (F designation in Table 3), the solid solution aging specimen (T6 designation in Table 3), the completely annealed specimen (O designation in Table 3), and the Al-Si system, the Al-Si-Cu system, and Al-Si-Cu-B system, with a lattice conductivity (λ_g_) of 42.3 W/(m·K) and apparent Lorentz constant (L) of 1.86 × 10^−8^. Thus, the thermal conductivity of any Al-Si-Cu alloy can be calculated at room temperate using:

(3) λ=5.45σ+42.3W/(m·K)

### 4.2. Trace Elements after Treatment by Al-B

Nearly 90% of the volume percentage of the Al-Si-Cu alloy is the Al phase. The trace elements come from the pure aluminum ingots and play an important role on the conductivity of the Al-Si-Cu alloys. Usually, there are several hundred ppm of Ti, Mn, V, and Cr in the pure aluminum ingots, these are included in the as-cast Al-Si-Cu alloy. Some of these elements may react with Si, Cu, and Fe, and form intermetallic compound. However, most remain in solid solution in the Al crystal lattice and significantly decrease the conductivity. The most efficient way to eliminate Ti is to add B element into melt, to allow Ti solute to react to form Al_3_Ti and TiB_2_ and to remove Ti from the Al crystal lattice.

Murty et al. [29,32] found that the Al–1Ti–3B master alloy (to achieve 0.03%B) was a good grain refiner for the Al–7%Si alloy and better than Al–5Ti–1B (to achieve 0.03% Ti). Sigworth and Guzowski [33] showed that Al–3Ti–3B master produced an equivalent grain size at addition levels of 50% to 75% of those required with the Al–5Ti–1B master alloy, with no fading during a holding period. This research indicated that B played a critical role in the formation of Al_3_Ti particles in the Al-Si alloy.

Kori [32] indicated that Ti reacts with Si and Al to form complex Se particles and aluminide particles containing Ti. This indicates that Ti is partially consumed by the formation of the silicon particles. However, the remaining Ti solute cannot form Al_3_Ti particles efficiently even in the presence of B, because of poisoning. Increasing the addition level of the Al-5Ti-B master alloy can overwhelm the poisoning influence in the high Si containing Al-Si system, but this increases the Ti content. In contrast, Kori [32] indicated that the Al-B master alloy produced excellent grain refinement of the Al-7%Si alloy as did the Al-Ti-B master alloy with high B content.

Clearly, increasing the Ti content by increasing Al-5Ti-B master alloy addition will overwhelm the Ti consumption of the Si particles and Si-enriched aluminide particle. However, it is difficult to understand how a certain amount of Al-B master alloy can have a much better effect on grain refinement than the Al-3Ti-3B master alloy. Ólafsson et. al. [34] showed that B has a greater binding force with Ti than with Si. Therefore, the coexistence of Si, B and Ti in Al matrix produces the following phases: Al, Si, TiB_2_, and AlB_2_. The grain refining of the Al-7%Si alloy by the Al-B master could occur by the following two possible mechanisms: (i) AlB_2_ particles provide good heterogeneous nucleation, and (ii) B helps Ti to form Al_3_Ti and TiB_2_ compounds, which provide nucleation. However, Kori [32] ignored the fact that Al-7%Si alloy was made from industrial-grade pure aluminum ingots (99.7%) with hundreds ppm of Ti, Mn, Cr, and V, so they ignored an important factor of the trace element when they investigated the influence of B.

The following research was carried out in order to investigate (i) whether a small amount of B can react with the trace Ti, Mn, V and Cr, (ii) how B increases the conductivity of the Al-Si-Cu alloy, and (iii) how B refines the primary Al phase.

**Figure 12:**
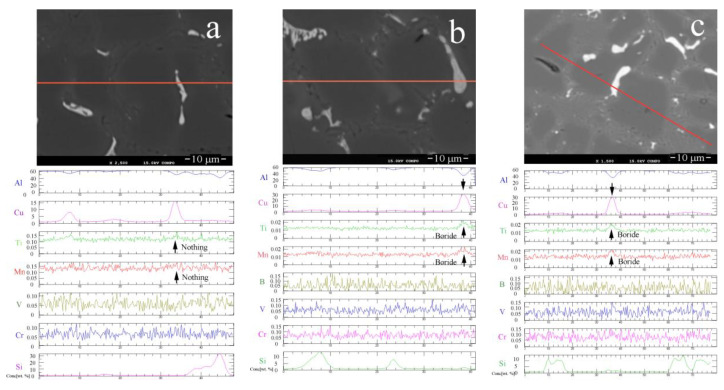
The line-scans of the major elements and trace elements measured byaJXA-8230 EMPA system for Al-9.8%Si-2%Cu containing: (**a**) no B; (**b**) 0.02% B; and (**c**) 0.05% B.

Figure 12 show the distribution of Al, Cu, Ti, Mn, V, Cr, and Si in Al-9.8%Si-2%Cu measured using a JXA-8230 EMPA system. Figure 12a presents the plots for Al-9.8%Si-2%Cu without B. Figure 12b presents the plots for Al-9.8%Si-2%Cu with 0.02% B. Figure 12c presents the plot for Al-9.8%Si-2%Cu with 0.05% B. The plots of the trace elements indicate that both the primary Al phase and the eutectic Al phase contain the Ti, Mn, V, and Cr elements. The Al-9.8%Si-2%Cu alloy not containing B (i.e., not treated with the Al-B master alloy) contained these transition group elements evenly distributed in the primary Al phase and in the eutectic Al phase. In contrast, the Al-9.8%Si-2%Cu alloy containing B had Ti and Mn peaks, that seemed to co-exist with the Cu-rich zone. This occurred in both the alloys of 0.02% B and 0.05% B, as shown in Figure 12b,c. Because B has a small atomic weight, no aggregation of B element was detected. In addition, there was no Ti or Mn aggregation in the Si crystals.

**Figure 13:**
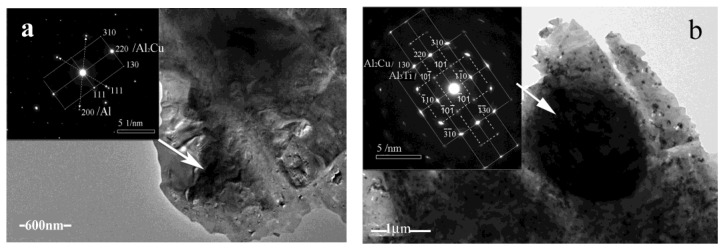
TEM photographs of typical Al_2_Cu particles in ADC12 alloys observed by a Tecnai G2 20ST TEM system: (**a**) a Al_2_Cu particle that did not contain Al_3_Ti particles for a B free alloy, and (**b**) a Al_2_Cu particle containing Al_3_Ti particles for an alloy containing B.

TEM observation was carried out by a Tecnai G2 20ST TEM system in order to investigate the role played by B on the aggregation of Ti and Mn in the Cn-enriched particles. Figure 13a presents a typical Cu-enriched particle in a specimen without B; the diffraction patterns are mainly from the Al_2_Cu particle and the Al matrix. Figure 13b presents a typical Cu-enriched particle in a specimen containing B; (i) the bright diffraction patterns indicate that the particles are Al_2_Cu, (ii) the relatively dim diffraction spots indicate Al_3_Ti, and (iii) there seems to be a co-lattice relationship between the Al_2_Cu particles and the Al_3_Ti particles.

Consider that (i) TiB_2_ is critical for the formation of Al_3_Ti so that Al_3_Ti particle cannot exist in molten aluminum without TiB_2_ particles and (ii) TiB_2_ particles and Al_3_Ti particle have a co-lattice. It is reasonable that B reacted with Ti and Mn forming borides, and promoted the formation of Al_3_Ti particles. For the Al_2_Cu and Al_3_Ti particles in a co-lattice, when the Al_3_Ti particle nucleated the Al phase, the Al_3_Ti particle promoted the deposit of the Al_2_Cu particle. These reactions absorbed part of the transition elements from the Al matrix, decreased the scattering of electrons, and increased the conductivity of the Al-9.8%Si-2%Cu alloys. Ti was not found in the Si particles. This result agreed with Gröbner’s calculation [35].

Figure 9 indicates that the Al-9.8%Si-2%Cu containing B has a crystal lattice constant much closer to the pure Al crystal lattice constant than the Al-9.8%Si-2%Cu without B. This explains why the Al-B master alloy increases conductivity. Figure 9 also indicates that the optimal B content is 0.02%. Even with Ti, Mn, V, and Cr, further increasing B content did not increase the conductivity of Al-9.8%Si-2%Cu. This can be explained by the Al-Ti-B ternary phase diagram [36]. When B is less than 0.02%, B completely reacted with the transition element. Increasing the B content increases the withdrawal of the transition elements from the Al base; however, when B content is more than 0.02%, B dissolves in the Al base, and decreases conductivity. Instead of withdrawing more transition element from the Al base, B itself dissolves in the Al base and distorts the crystal lattice. Therefore, 0.02% B is the optimal content for the conductivity of Al-9.8%Si-2%Cu.

The addition of the Al-B master alloy also refined the primary Al grain size, in addition to the positive influence on the conductivity. The mechanism of grain refinement was investigated by Dahle [37,38] who showed that the Al-Ti-B master alloy with a lower Ti/B ratio produced better grain refining. Figure 8 shows the backscattered electron images of the microstructure of Al-9.8%Si-2%Cu with and without B. The B refined the Al-9.8%Si-2%Cu with trace amounts of Ti, V, Cr, and Mn, in agreement with the research of Dahle [37,38]. It is proposed that the removal of the Ti, V, Cr, and Mn from the Al phase should decrease the hardness of the Al matrix of the Al-9.8%Si-2%Cu alloy. However, the Al-9.8%Si-2%Cu alloys containing B had the same strength and hardness as the Al-9.8%Si-2%Cu without B. This indicated that the solid solution strengthening by Ti, V, Cr, and Mn was not significant, and that the grain refinement compensated for the decrease of solution strengthening.

When the B was added, α-Al primary phase transferred from long dendritic particles to fine and equiaxed rosette-like particles, as shown in Figure 8. Based on Al-Si phase diagram, the finer the α-Al primary phase particles, the higher level of Al element and the lower level of Si element, which means more volume percentage of eutectic structure occurs around the fine and equiaxed α-Al particles.

### 4.3. Distorsion of Aluminum Crystal

Solute element in pure Al matrix changes the lattice constant of Al crystal. The more the deviation of the radius of the solute element from the radius of Al, the more severe the lattice is distorted. The radius of the elements is listed in Table 4. Cu, V, Cr, and Mn increase the distortion density of pure aluminum by a higher level than Ti.

The lattice constant of Al without distortion is 4.048 Ǻ. The solution of Si in Al is very small. Therefore, Si does not change the lattice constant significantly. The adding of Cu improves the mechanical properties of ADC12; however, it decreases the lattice constant of Al crystal from 4.048 Ǻ to 4.045 Ǻ, and deteriorate the conductivity of ADC12. Without B adding, the Ti, V, Cr, Mn, and other elements change the lattice constant of Al to 4.043 Ǻ. After B adding, the Ti, V, Cr, and Mn composite form boride with B. The lattice constant increased to 4.047 Ǻ, which is near the pure Al lattice constant. If excessive B element solute in Al crystal, the lattice distortion deteriorates again.

## 5. Conclusions

1. The conductivity of ADC12 between 7~12.8% Si decreased linearly with increasing volume fraction of the Si phase attributed to a linear decrease of the volume of the more conductive Al phase through a rule of mixtures. Si has little influence on the lattice constant.
2. The conductivity of ADC12 decreased, and the degree of Al-Si lattice distortion increased, with increasing Cu content, between 0~3%. The influence of Cu could be deduced from the Al lattice constant deviation.
3. The conductivity of ADC12 sharply increased with increasing B content, reached the peak at 0.02% B and thereafter decreased somewhat. The mechanism was that B reacted with the transition element in the Al phase to form boride, decreasing the transition element concentration in the Al lattice, and decreasing the lattice constant.
4. In Al-Si-Cu system, the thermal conductivity, λ, was related to the electrical conductivity, σ, by means of $\;\lambda =\mathrm{LT}\sigma +\lambda_{g}$, where L is the apparentLorentz constant, 1.86 × 10^−8^; T is the absolute temperature, 293 K; λ_g_ is the lattice conductivity, 42.3 W/(m·K).

## Figures and Tables

**Table 1:** The most commonly used commercial high conductivity Al-Si Alloys.

Alloys	Si	Cu	Heat Diffusion Coefficient.	Capacity	Thermal Conductivity	Electrical Conductivity
	wt.%	wt.%	mm^2^/s	J/g·K	W/(m·K)	MS/m
AlSi12 (Fe)	12	-	63.361	0.872	147.52	19.45
A360	9.5	-	60.968	0.871	142.316	19.32
AlSi10Mg (Fe)	10	-	63.475	0.876	149.575	19.91
ADC12	11	2	56.68	0.856	131.484	15.66
ADC12 (high Cu)	11	3	54.646	0.854	126.003	15.03
A383	10.5	2	56.104	0.867	130.847	16.14
A413.0	10.5	-	62.715	0.877	147.403	16.99
A380	8.5	2	56.803	0.859	130.767	16.19
AlSi9Cu3	9	3	54.307	0.852	125.853	15.03
AlSi8Cu3 (Fe)	8	3	52.804	0.85	121.634	15.33
AlSi12Cu1Fe	12	2	61.218	0.85	141.016	16.14

**Table 2:** Compositions of pure Al ingot and Al-9.8Si-2Cu with/without Al-B master alloy.

	Si/%	Fe/%	Cu/%	Mn/ppm	Ga/ppm	V/ppm	Cr/ppm	Ti/ppm	B	Al
AA1070	0.69	0.17	-	31	103	38	25	40	-	Bal.
Si (441)	99.16	0.39	-	51	-	10	9	13		0.37
Al-9.8Si-2Cu	9.83	0.18	2.11	28	109	34	30	36	-	Bal
Al-9.8Si-2Cu-B	9.86	0.16	2.03	30	88	36	28	41	50	Bal

**Table 3:** Conductivities and mechanical properties of the specimens with different compositions, in which F is as-cast, T6 is solid solution aging and O is complete annealing.

AlloyNo.		Nominal Composition (wt.%)			State	Electrical Conductivity	Tensile Strength	Elongation	Thermal Conductivity
	Si	Cu	B	Al		(MS/m)	(MPa)	(%)	W/(m·K)
1	7.0	0	0	Bal	F	19.83	150	5.5	147.9
2	9.0	0	0		F	19.32	160	3.5	133.9
3	9.8	0	0		F	19.14	174	2.7	130.0
4	10.8	0	0		F	18.76	190	5.0	121.47
5	11.8	0	0		F	18.66	218	7.2	119.3
6	12.8	0	0		F	18.48	220	2.3	118.5
7	9.8	1	0		F	18.3	194	1.7	116.94
8	9.8	2	0		F	18.12	223	4.2	118.9
9	9.8	3	0		F	17.90	214	3.3	113.5
10	9.8	2	0.01		F	19.00	143	1.9	140.0
11	9.8	2	0.02		F	19.22	226	2.3	146.2
12	9.8	2	0.03		F	18.78	161	1.8	138.8
13	9.8	2	0.05		F	18.70	194	4.1	138.4
14	9.8	2	0.02		T6	22.14	221	1.8	166.7
15	7.0	0	0.02		F	22.57	130	2.8	162.7
17	7.0	0	0.02		O	30.23	130	9.3	209.5

**Table 4:** Compositions of pure Al ingot and Al-9.8Si-2Cu with/without Al-B master alloy.

Atomic Radius	Al	Si	Cu	Ti	V	Cr	Mn	B
Ǻ	1.43	1.34	1.28	1.45	1.35	1.27	1.32	0.95

## Data Availability

All data included in this study are available upon request by contact with the first author (zyang@csu.edu.cn).
